# Primary cutaneous anaplastic large‐cell lymphoma with marked spontaneous regression of organ manifestation after SARS‐CoV‐2 vaccination

**DOI:** 10.1111/bjd.20630

**Published:** 2021-12-01

**Authors:** T. Gambichler, S. Boms, S. Hessam, I. Tischoff, A. Tannapfel, T. Lüttringhaus, J. Beckman, R. Stranzenbach

**Affiliations:** Skin Cancer Center Department of Dermatology Ruhr‐University Bochum BochumGermany; Department of Dermatology Christian Hospital Unna UnnaGermany; Department of Dermatology Christian Hospital Unna UnnaGermany; Institute of Pathology Ruhr‐University Bochum Bochum Germany; Institute of Pathology Ruhr‐University Bochum Bochum Germany; Haemato‐Oncological Outpatient Clinic Christian Hospital Unna UnnaGermany; Department of Radiology Christian Hospital Unna Unna Germany; Skin Cancer Center Department of Dermatology Ruhr‐University Bochum BochumGermany

Dear Editor, Primary cutaneous anaplastic large‐cell lymphoma (pcALCL) belongs to the primary cutaneous CD30+ T‐cell lymphoproliferative disorders.^1^ Systemic involvement is relatively rare and pcALCL is generally confined to the regional lymph nodes.^1^ In this research letter, we describe a patient with recurrent pcALCL and diffuse lung manifestation that spontaneously regressed after one SARS‐CoV‐2 vaccination.

We report a 57‐year‐old male patient with a 10‐year history of biopsy‐proven [CD30+, CD3+, CD4+, anaplastic lymphoma kinase (ALK)−, clonal T‐cell receptor gamma‐chain rearrangement] pcALCL with frequent local relapses predominantly affecting his scalp and neck. Moreover, the patient had a history of suspicious waning and waxing cervical lymph nodes (partly biopsy‐proven) over the last 5 years. Pretreatments included methotrexate, brentuximab, gemcitabine and radiotherapy. The last treatment (radiotherapy) of cutaneous lesions (Figure 1a–d) was performed in February 2021, resulting in complete remission. At the end of March 2021, ultrasound again revealed a pathologically enlarged cervical lymph node (Figure 1e). Thoracic computed tomography (CT) showed innumerable bilateral pulmonary nodules that were suspicious for new pcALCL manifestation or second malignancy (Figure 1g). Bone marrow was not involved. A lung wedge resection was scheduled, which included a histological confirmation by a haematopathology reference centre. Histopathology of several lesions of the lung revealed infiltrates of Hodgkin/Reed–Sternberg‐like cells that were positive for CD30, CD3, CD4, TGP and CD15 and negative for Bcl‐6, epithelial membrane antigen and ALK‐1. On multiplex polymerase chain reaction, there was a monoclonal T‐cell receptor gamma‐ (tube A, 244 bp) and beta‐chain (tube A, 254 bp; tube B, 255 bp) rearrangement in the lung tissue, which was exactly the same pattern as observed in biopsies of skin lesions 5 years and 9 years previously. Rechallenge with brentuximab was discussed by the interdisciplinary tumour board. A few days before initiation of brentuximab therapy, the patient noticed shrinking of the suspicious cervical lymph node, which was confirmed by means of ultrasound (Figure 1f). Moreover, thoracic CT revealed an almost complete resolution of the diffuse lung lesions (Figure 1h). Importantly, 1 week before pcALCL restaging, the patient had received his first COVID‐19 vaccination (Comirnaty®, BioNTech/Pfizer, Mainz, Germany).

**Figure 1:**
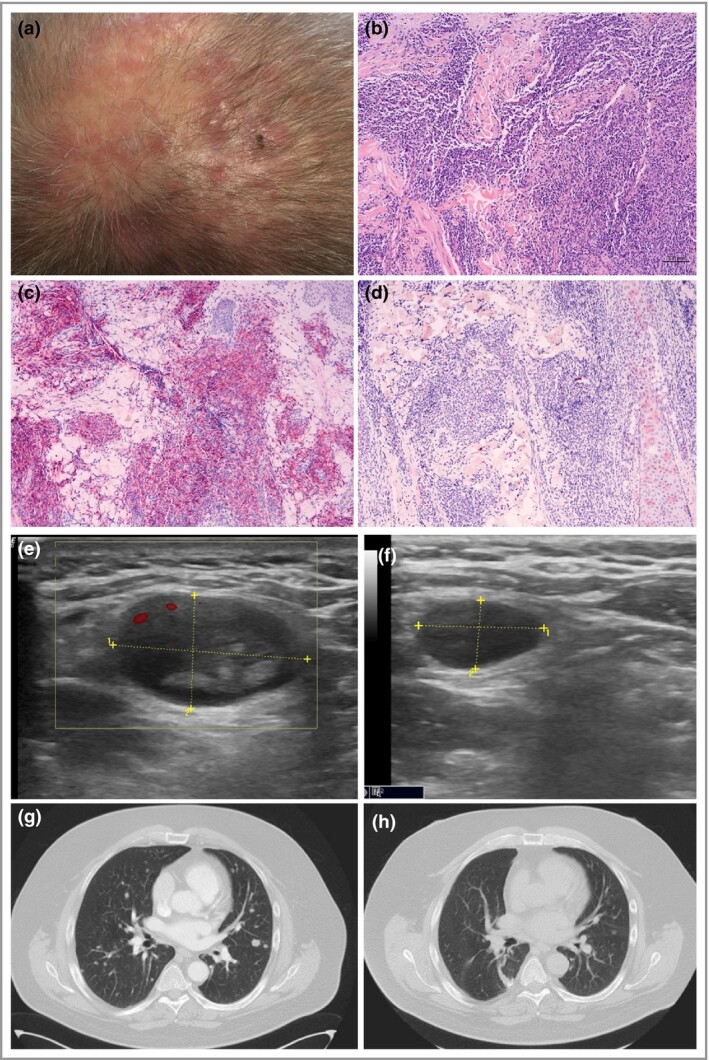
(a) A male patient is shown with recurrent primary cutaneous anaplastic large‐cell lymphoma (pcALCL) on the scalp. (b) Haematoxylin & eosin histology revealed dense dermal infiltrates with large lymphocytes (scale bar = 100 µm) showing (c) strong CD30 positivity and (d) CD15 negativity on immunohistochemistry. (e) Ultrasound revealed a pathologically enlarged cervical lymph node (1 = 2·2 cm, 2 = 1·29 cm). (g) Thoracic computed tomography showed diffuse bilateral pulmonary disease with numerous tumour nodules. Shortly before treatment initiation the patient had received his first SARS‐CoV‐2 vaccination. (f) A few days later, the size of the lymph node was significantly decreased (1 = 1·23 cm, 2 = 0·71 cm) and pulmonary manifestation had almost completely spontaneously regressed (h).

pcALCL is a rare lymphoma with an excellent prognosis as indicated by a 10‐year survival rate of about 90%. However, the recurrence rate of pcALCL is high. Notably, 10–42% of patients diagnosed with pcALCL may experience spontaneous remission.^1^ We detected identical clonal T‐cell receptor gamma‐chain rearrangements in lung lesions and previous skin lesions, together proving that the pulmonary lesions very likely originated from pcALCL. Eberle *et al*.^2^ demonstrated that systemic manifestations of pcALCL frequently show Hodgkin/Reed–Sternberg‐like infiltrates and that the coexpression of CD30 and CD15 in these cases may result in a mistaken diagnosis of classical Hodgkin disease.^2^ Diffuse pulmonary disease stemming from pcALCL, as described in the present letter, is certainly unusual, as is spontaneous regression of such a tumour load, even though pcALCL is often associated with spontaneous regression as mentioned before.^1^, ^2^

The most frequently reported triggers of spontaneous remission of malignancies include surgical trauma, infections and vaccines. Several vaccines, including the vaccines for smallpox, tetanus‐diphtheria‐pertussis, rabies and the Bacillus Calmette–Guérin vaccine, have previously been reported to be associated with spontaneous cancer remission. Accordingly, medical research has focused on cancer microbial vaccine immunotherapy.^3^, ^4^ Our patient received the COVID‐19 vaccination 1 week before the detection of the almost complete resolution of his untreated lymphoma manifestations. After his COVID‐19 vaccination, he noticed that the suspicious cervical lymph node regressed, indicating that the remission process likely began after the vaccination. Hence, the temporal sequence strongly suggests, but does not prove, that the SARS‐CoV2 vaccine was the causal factor of the marked spontaneous regression of organ manifestation observed in the present case.

Dotan *et al*.^5^ proposed that the most likely mechanism to have the potential for contributing to the development of autoimmunity in COVID‐19 is the capability of SARS‐CoV‐2 to overstimulate the immune system in addition to the molecular mimicry between self‐components of the host and the viral components. There is growing evidence that autoimmunity owing to COVID‐19 infection can also be triggered by SARS‐CoV‐2 vaccines.^5^–^7^ The combination of a genetically predisposed individual with an overstimulated immune system owing to SARS‐CoV‐2 vaccination may trigger autoimmune phenomena or diseases such as myasthaenia gravis, Henoch–Schönlein purpura or Rowell's syndrome.^5^–^7^ Thus, we speculate that SARS‐CoV‐2 vaccines have the potential not only to trigger autoimmunity, but also to enhance antitumour responses owing to overstimulation of the immune system. In contrast, Brumfield *et al*.^8^ recently reported on the recurrence of a primary cutaneous CD30‐positive lymphoproliferative disorder following COVID‐19 vaccination.

In conclusion, we observed marked spontaneous regression of organ manifestation in a patient with recurrent pcALCL following their first SARS‐CoV‐2 vaccination. However, as up to 42% of pcALCL cases may show spontaneous regression, we cannot fully exclude the possibility that this occurred in the present case.

## Acknowledgments

Open Access funding enabled and organized by Projekt DEAL.

## Author Contribution

**Thilo Gambichler:** Conceptualization (lead); Formal analysis (equal); Supervision (equal); Validation (equal); Visualization (equal); Writing‐original draft (equal); Writing‐review & editing (equal). **Stefanie Boms:** Conceptualization (equal); Data curation (equal); Resources (equal); Writing‐original draft (equal); Writing‐review & editing (equal). **Schapoor Hessam:** Data curation (equal); Writing‐original draft (equal); Writing‐review & editing (equal). **Iris Tischoff:** Investigation (equal); Validation (equal); Visualization (equal); Writing‐review & editing (equal). **Andrea Tannapfel:** Investigation (equal); Methodology (equal); Validation (equal); Visualization (equal); Writing‐review & editing (equal). **Timo Lüttringhaus:** Formal analysis (equal); Resources (equal); Writing‐review & editing (equal). **Jaques Beckman:** Formal analysis (equal); Visualization (equal); Writing‐review & editing (equal). **Rene Stranzenbach:** Formal analysis (equal); Validation (equal); Writing‐review & editing (equal).
